# Small Body, Large Chromosomes: Centric Fusions Shaped the Karyotype of the Amazonian Miniature Fish *Nannostomus anduzei* (Characiformes, Lebiasinidae)

**DOI:** 10.3390/genes14010192

**Published:** 2023-01-11

**Authors:** Renata Luiza Rosa de Moraes, Francisco de Menezes Cavalcante Sassi, Manoela Maria Ferreira Marinho, Petr Ráb, Jorge Ivan Rebelo Porto, Eliana Feldberg, Marcelo de Bello Cioffi

**Affiliations:** 1 Departamento de Genética e Evolução, Universidade Federal de São Carlos (UFSCar), Rodovia Washington Luiz Km. 235, C.P. 676, São Carlos 13565-905, SP, Brazil; 2 Departamento de Sistemática e Ecologia, Universidade Federal da Paraíba, Cidade Universitária, Castelo Branco, João Pessoa 58051-900, PB, Brazil; 3 Laboratory of Fish Genetics, Institute of Animal Physiology and Genetics, Czech Academy of Sciences, Rumburská 89, 277 21 Liběchov, Czech Republic; 4 Laboratório de Genética Animal, Coordenação de Biodiversidade, Instituto Nacional de Pesquisas da Amazônia, Av. André Araújo 2936, Petrópolis, Manaus 69067-375, AM, Brazil

**Keywords:** neotropical fishes, 2n reduction, karyotype, chromosomal rearrangements

## Abstract

Miniature refers to species with extraordinarily small adult body size when adult and can be found within all major metazoan groups. It is considered that miniature species have experienced severe alteration of numerous morphological traits during evolution. For a variety of reasons, including severe labor concerns during collecting, chromosomal acquisition, and taxonomic issues, miniature fishes are neglected and understudied. Since some available studies indicate possible relationship between diploid chromosome number (2n) and body size in fishes, we aimed to study one of the smallest Neotropical fish *Nannostomus anduzei* (Teleostei, Characiformes, Lebiasinidae), using both conventional (Giemsa staining, C-banding) and molecular cytogenetic methods (FISH mapping of rDNAs, microsatellites, and telomeric sequences). Our research revealed that *N. anduzei* possesses one of the lowest diploid chromosome numbers (2n = 22) among teleost fishes, and its karyotype is entirely composed of large metacentric chromosomes. All chromosomes, except for pair number 11, showed an 18S rDNA signal in the pericentromeric region. 5S rDNA signals were detected in the pericentromeric regions of chromosome pair number 1 and 6, displaying synteny to 18S rDNA signals. Interstitial telomeric sites (ITS) were identified in the centromeric region of pairs 6 and 8, indicating that centric fusions played a significant role in karyotype evolution of studied species. Our study provides further evidence supporting the trend of diploid chromosome number reduction along with miniaturization of adult body size in fishes.

## 1. Introduction

Miniaturization refers to species with exceptionally small adult body size that have experienced reduction and severe alterations of various morphological features. Miniature species are present and widespread across all major metazoan groups, including fishes [1,2,3]. It is considered that miniature fish species do not exceed 26 mm of standard body length (SL) and are frequently characterized by morphological modifications of the latero-sensory canal system of head and body, lower number of fin rays and body scales, as well as less structured bones [4,5,6]. With more than 200 described cases, the Neotropical region harbors the highest number of miniature fish species around the world [4,5,7,8,9,10,11,12,13]

When compared to cartilaginous fishes and mammals, teleosts have experienced a higher rate of chromosomal rearrangements and a faster evolution of protein sequences (reviewed in [14]). Such chromosomal rearrangements are known to disrupt some gene-linkage groups and may lead to alterations of the organism’s morphological traits, including body size [15]. In wasps, genes evolve faster in miniaturized species when compared to regular-sized ones [16]. In salamanders, smaller body size, slower cellular differentiation, less complexity of brain, and larger sensory organs are observed in species that are characterized by larger genomes [17,18]. Although evidence from several animal groups suggest that adult body sizes and genome organization are somehow linked [1,16,19,20], few studies have compared chromosome number and animals’ body size in the context of the karyotype evolution process, so far [19,20,21].

Lebiasinid fishes are representatives of Lebiasinidae family (Characiformes order) that are distributed across Central and South America. The family comprises 75 species, consisting of two subfamilies, namely: (1) Lebiasininae (*Derhamia*, *Lebiasina,* and *Piabucina*), with small to medium-sized species reaching 60–160 mm SL and (2) Pyrrhulininae (*Copeina*, *Copella*, *Nannostomus*, and *Pyrrhulina*), a clade containing small to miniature species, which attain body size up to 85 mm SL [8,21,22]. Among lebiasinids, pencil fishes (*Nannostomus* genus) represented by *N. anduzei, N. britski* and *N. minimus* are well known miniature species that reach maximum 24 mm SL [8,21]. They are popular as ornamental fishes traded for aquarists because of their bright coloration [21,23]. 

The small size of lebiasinid fishes hampered their cytogenetic investigations for many years [24,25,26]. However, recent progress in cytogenetic techniques has overcome this issue, broadening our knowledge of the structure and mechanisms of their karyotype evolution [23,27,28,29,30,31,32]. Species karyotyped to date by conventional and molecular techniques have revealed two main scenarios of chromosome evolution within the family [23,27,28,29,30,31,32]. The first one supposes an ancestral state of chromosome number (2n = 36) that is conserved among Lebiasininae species, represented by *Lebiasina* (*Lebiasina bimaculata*, *Lebiasina minuta, Lebiasina melanoguttata*). In turn, the second evolutionary scenario hypothesizes a large drop out of diploid chromosome number (2n = 22–32) in some Pyrrhulininae species, especially in *Pyrrhulina* and *Nannostomus* via Robertsonian fusions resulting in the presence of large metacentric chromosomes [23,29]. 

Due to considerable difficulties in chromosome analysis of pencil fishes, the majority of cytogenetic studies carried out are limited to Giemsa staining and other conventional banding methods. So far, the available data show large variation in their diploid chromosome number, ranging from 2n = 22 to 46 (reviewed in [26]). A recent conventional and molecular study on four *Nannostomus* species highlighted that their karyotype evolution was predominantly driven by substantial structural changes, with a relatively low divergence of their repetitive DNA content at the chromosomal level. Among pencil fishes, *N. unifasciatus* bears the lowest diploid chromosome number, which is also the smallest one among Neopterygian fishes [23].

In the present work, we aimed to broaden our understanding of karyotype evolution of small-sized fishes by examining one of the tiniest Neotropical fishes, the miniature *N. anduzei*, a species that does not surpass 16 mm SL [21]. For this purpose, we used both conventional (Giemsa staining, C-banding) and molecular cytogenetic approaches (FISH mapping of rDNAs, microsatellites, and telomeric sequences). The cytogenetic study performed by us provides new useful data on the possible association between the reduction of diploid chromosome number (2n) and body miniaturization in fish. The present results are part of our broader cytogenetics and cytogenomics research on Lebiasinidae fishes.

## 2. Materials and Methods

### 2.1. Samples and Chromosomal Preparation

**Figure 1:**
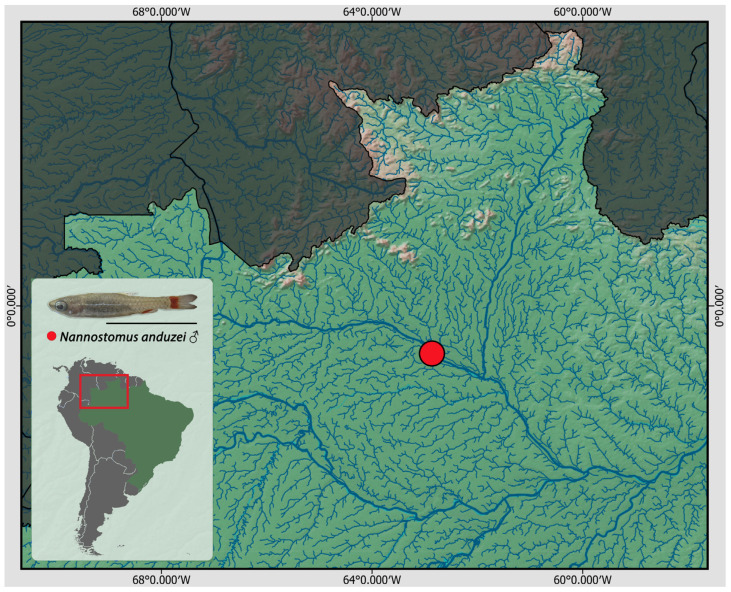
Collection site of *N. anduzei* in Barcelos—AM. Scale bar equals 1 cm.

Fourteen individuals of *N. anduzei* were collected in the middle part of Negro River at Zamula stream in Barcelos—AM (Figure 1). The sex of collected fish was determined based on the body dimorphism expressed by differential coloration and by direct observation of gonadal morphology. In order to collect tissues of liver, kidney, spleen, and gills, a stereomicroscope was used. Chromosome isolation from collected tissues was carried out in accordance with conventional air-drying technique [33]. Following the dissection, the complete fish bodies were preserved in 100% and deposited in the tissue bank of the Laboratório de Citogenética de Peixes of Universidade Federal de São Carlos (São Carlos—SP, Brazil) with the voucher numbers 23359, 23360, 23361, 23362, 23363, 23364, 23365, and 23366. All individuals were collected under the appropriate authorization of the Brazilian environmental agency ICMBIO/SISBIO (License number 48628-14) and SISGEN (A96FF09). The experiments followed ethical standards, and anesthesia in accordance with the Ethics Committee on Animal Experimentation of the Universidade Federal de São Carlos (Process number CEUA 1853260315).

### 2.2. Conventional and Molecular Cytogenetics

Constitutive heterochromatin was revealed by the application of a standard C-banding procedure [34]. The rDNAs, microsatellites, and telomeric sequences were mapped by fluorescence in situ hybridization (FISH) with high stringency conditions [35], where metaphase chromosomes were treated with RNAse A (40 μg/mL) for 1.5 h at 37 °C and the DNA was denatured in 70% formamide/2× SSC at 72 °C for 3 min. 20 μL of the hybridization mixture (2.5 ng/μL probes, 50% deionized formamide, 10% dextran sulfate) was then applied, and the hybridization process was performed overnight at 37 °C in a moist chamber. The first post-hybridization wash was performed with 1× SSC for 5 min at 65 °C in a shaker, followed by 4× SSC/Tween for 5 min at room temperature. Chromosomes were counterstained with DAPI with Vectashield (Vector Laboratories, Burlingame, CA, USA). The probes of ribosomal 5S and 18S rDNA were isolated from *Hoplias malabaricus* genome [36,37]. The 5S rDNA probe included 120 base pairs (bp) of the 5S rDNA gene coding region and 200 bp of non-transcribed spacer (NTS) [36]. The 18S rDNA probe was composed of a 1400-bp-long segment of the 18S rDNA coding region [37]. Both probes were directly labeled with the NickTranslation Mix Kit according to the manufacturer’s instructions (Jena Bioscience, Jena, Germany), the 18S rDNA probe with Atto488-dUTP and the 5S rDNA with Atto550-dUTP. Microsatellites were also mapped: The (CA)_15_, (GA)_15_, and (CGG)_10_ probes were directly labeled with Cy3 during the synthesis [38]. Telomeric regions were detected using the standard vertebrate telomere sequence (TTAGGG)n, obtained by PCR in the absence of a template according to [39] and later labeled with Atto550-dUTP with the Nick-Translation Mix Kit (Jena Bioscience, Jena, Germany).

### 2.3. Microscopic Analyses and Image Processing

To determine diploid number of chromosomes, karyotype structure, and FISH mapping results, at least 20 metaphase spreads per individual were examined. Images were captured with an Olympus BX50 microscope (Olympus Corporation, Ishikawa, Japan) equipped with a Teledyne Photometrics CoolSNAP CCD camera (Teledyne Photometrics, Tucson, AZ, USA), and processed with Image-Pro Plus 4.1 software (Media Cybernetics, Silver Spring, MD, USA). The chromosomes were classified as metacentric (m), submetacentric (sm), and acrocentric (a) according to Levan and colleagues’ nomenclature [40].

## 3. Results

**Figure 2:**
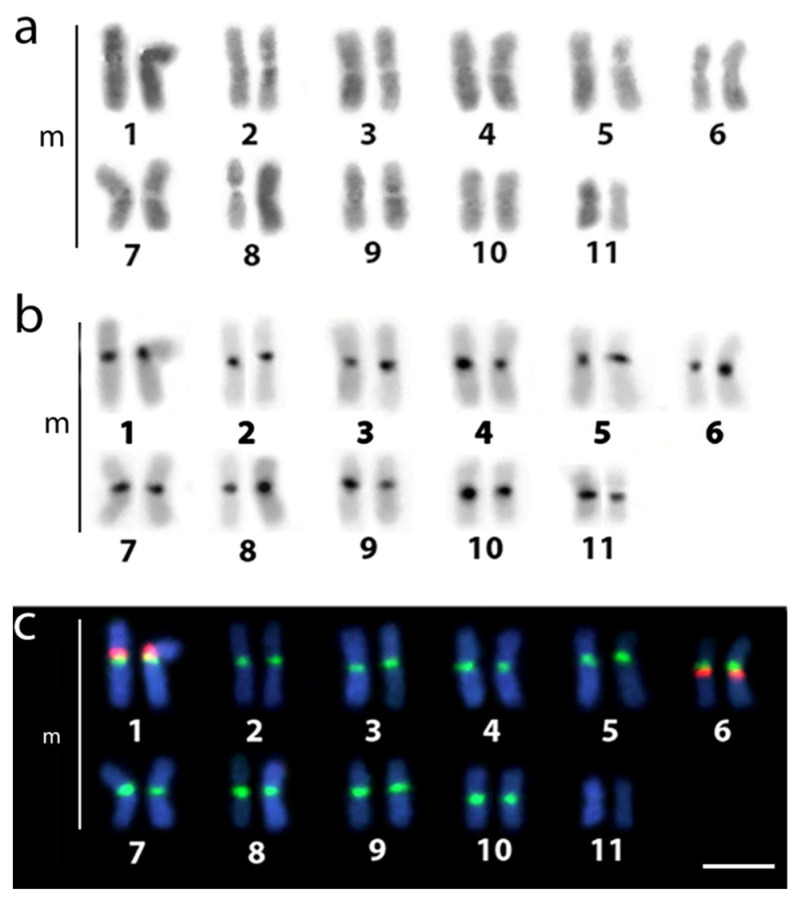
Karyotypes of *N. anduzei* subjected to sequential Giemsa staining (**a**), C-banding (**b**), and FISH (**c**) with 5S (red) and 18S (green) rDNA probes. Scale Bar = 10 µm.

The diploid chromosome number in all analyzed fish was 2n = 22. The karyotype was exclusively composed of large metacentric chromosomes, with C-positive heterochromatin blocks found in the pericentromeric region of all chromosomes (Figure 2a,b). Except for pair number 11, all chromosomes displayed an 18S rDNA signal in the pericentromeric region, which was found in synteny with 5S rDNA localized on chromosomal pairs number 1 and 6 (Figure 2c).

**Figure 3:**
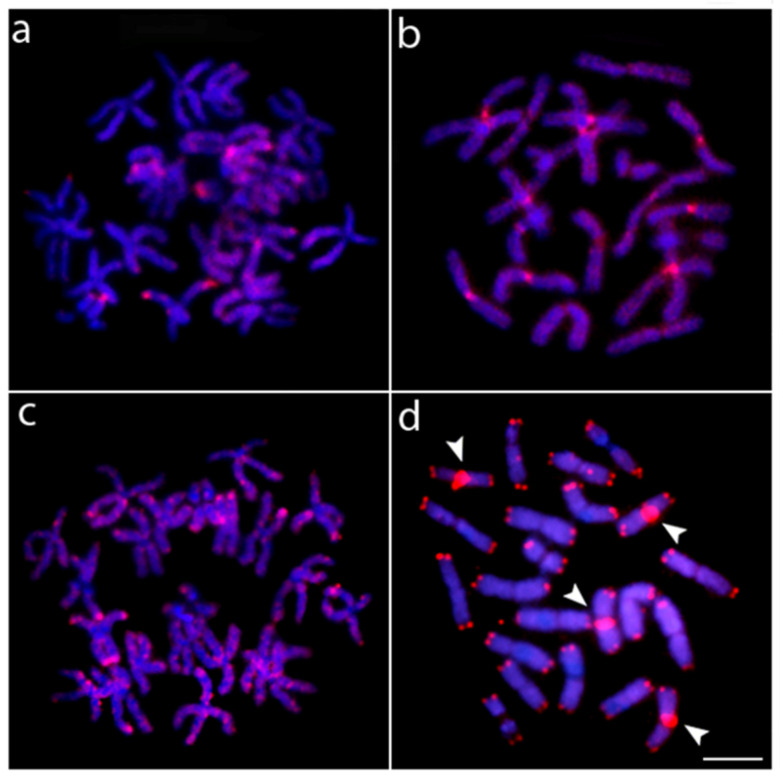
Metaphase chromosomes of *N. anduzei* after FISH hybridization with (**a**) (GA)_15_; (**b**) (CGG)_10_; (**c**) (CA)_15_ microsatellite DNA probes, and (**d**) (TTAGGG)n sequences. Arrowheads indicate the interstitial telomeric sites (ITS) observed on pairs 4 and 6. Scale Bar = 10 µm.

Chromosome mapping with the (CA)n, (GA)n, and (CGG)n microsatellite probes displayed a similar pattern for males and females. The microsatellites (CA)n and (GA)n displayed signals in the terminal region of almost all chromosomes, whereas (CGG)n motifs are found in the centromeric region of nearly all chromosomes (Figure 3). Telomeric sequences (TTAGGG)n were found in the terminal regions of all chromosomes. Telomeric interstitial sites (ITS) were found in four chromosomes of pairs 6 and 8 (Figure 3).

## 4. Discussion

### 4.1. Chromosomal Characteristics of Nannostomus Anduzei

To date, only 7 out of 20 described *Nannostomus* species have been karyotyped ([14,26] and present study). Among them, *N. anduzei* (present study) and *N. unifasciatus* [23,26] are the species that have the lowest diploid numbers ever documented for teleost fishes [26], with karyotype exclusively composed of large metacentric chromosomes (Figure 1). Due to limited karyotypic data, the possible mechanisms of the decreased diploid number in *Nannostomus* remains a mystery. It is considered that observed large diploid chromosome number decrease in *N. anduzei* (2n = 22) and *N. eques* (2n = 36) when compared to other closely related pencil fishes, i.e., *N. backfordi* (2n = 44), *N. marginatus* (2n = 42), *N. unifasciatus* (2n = 40) and *N. harrisoni* (2n = 40) is the result of centric fusion events of Robertsonian type that most probably played a substantial role in genome evolution of Lebiasinid fishes [41,42]. Robertsonian fusions are one of the most prevalent chromosomal rearrangements observed during the karyotypic evolution of vertebrates (reviewed in [43]). This type of rearrangement can take place in three ways, namely: (i) The formation of dicentric chromosomes with loss of telomeric sequence [44], (ii) chromosome fission events [45,46], and (iii) chromosome fusion by retaining (and inactivating) the telomeric region [43]. However, telomere sequences can be further accumulated in the pericentromeric region of rearranged chromosomes, resulting in an interstitial telomeric sequence (ITS), as seen here in *N. anduzei* as well as in other vertebrates (e.g., [47,48,49,50]). Several fish species, including, e.g., *Erythrinus erythrinus* (Characiformes), *Hoplias malabaricus* (Characiformes), *Rhodeus ocellatus kurumeus* (Cypriniformes), and *Harttia carvalhoi* (Siluriformes), have prominent ITS [51,52,53,54]. However, the ITS are often lost due to rearrangement’s repair mechanisms [50], as recorded other Neopterygian fishes [29,55,56,57,58,59].

It is worth noting that, from a cytogenetic standpoint 18S and 5S rDNA regions can also be involved in centric fusion events because of their susceptibility to promote double-stranded DNA breakage (e.g., [60,61,62]). Available studies indicate that the terminal location of the 18S rDNA loci on the chromosomes, as well as terminal and interstitial localization of 5S rDNA regions are typical characteristics in all lebiasinid species [27,28,29,30,31,32,63]. Furthermore, some members of the family have genomes with multiple rDNA sites [27,28,29,30,31,32,63], including the synteny of both 18S and 5S rDNAs localization [27,28,29,30,31,63]. The presence of multiple rDNA sites differs from the usual pattern of the fish genomes, where most species have a single pair of 5S and/or 45S rDNA sites [64,65]. Multiple rDNA loci may thus imply high dynamics of genome rearrangements probably due to ongoing interspecific divergence or fast fixation caused by genetic drift in small populations [66,67,68]. Some fish species, such as the walking catfish *Clarias batrachus*, have 32 out of the 52 chromosomal pairs bearing rDNA sequences in the pericentromeric region, including a synteny of both 5S and 18S rDNAs [69]. In cichlid *Cichlasoma amazonarum* (Perciformes, Cichlidae—[70]) and the bank voles *Myodes glareolus* (Rodentia, Cricetidae—[71]), the spreading of 18S rDNA was associated with the exposure to environmental contaminants since this sequence is a component of heterochromatin and acting as a centromere protector [71,72]. *N. anduzei*, as well as some Pyrrhulina spp. and *L. minuta*, have multiple 18S rDNA loci [27,28,29,30,31,63], which distinguishes them from other previously studied *Nannostomus* species [73]. Interestingly, *N. anduzei* differs from its congeners by having 18S/5S rDNA synthetic association in pairs 1 and 6 (Figure 2c). Despite these findings, a clear relationship between rDNA-bearing chromosomes and the central fusion process cannot be confirmed.

Microsatellites are repeating sequences that, like rDNA, may be applied to examine fish genomes in an evolutionary context [74]. Microsatellite clustering can assist in tracking the level of subchromosomal dynamics and potentially give vital information on sex chromosome differentiation processes (e.g., [38,75,76,77]). Performed by us, microsatellite DNA chromosomal mapping in the Nannostomus aduzei revealed comparable to other Lebiasinidae species’ genomic microsatellite DNA distribution [27,28,29,30,31,32,63]. The (CA)_15_ motif shared a similar distribution pattern to *Pyrrhulina semifasciata* [28] and *Pyrrhulina obermulleri* [29], appearing almost exclusively in telomeric regions, whereas (GA)_15_ was more dispersed throughout chromosomes and exhibited a higher affinity in telomeric regions. Finally, (CGG)_10_ patterns showed a predilection for centromeric regions, overlapping with the location of rDNAs, as also observed in other species in the family [29]. Correlations between (CGG)_10_ and 18S rDNA distribution have been reported in several fish species, including, e.g., *Hepsetus odoe* [78], *L. bimaculata* [30], and several Siluridae species [79].

### 4.2. Chromosomal Reduction and Miniaturization: Cause-Effect Relationship or an Indirect Association?

For a broad range of reasons, miniature fishes are neglected and understudied since their study presents significant challenges, including difficulties in their sampling [13], taxonomic identification [40], and chromosomal preparation [23,29,30,31,32], among others. Irrespective of these, few available studies demonstrate notably lower diploid chromosome numbers of miniature fish species compared to others [19,20]. However, some species do not fit this view. For example, *N. unifasciatus,* that can reach up to 38.5 mm SL, also holds the same reduced karyotype characteristics [23]. The cut-off limit of 26 mm SL for “miniatures” may not include all species with morphological and genetic alterations related to body-size reduction.

**Figure 4:**
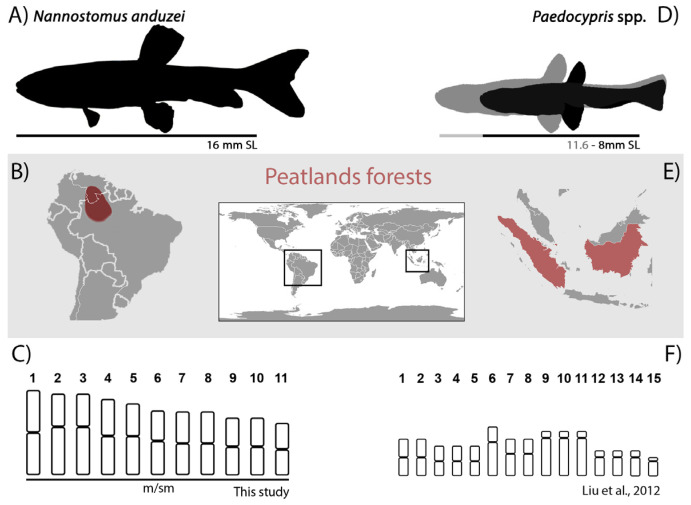
The main similarities between *N. anduzei* ((**A**–**C**) in this research) and *Paedocypris* spp. ((**D**–**F**)—[19]). (**A**,**D**) Representation of an adult specimen of *N. anduzei* with SL = 16 mm and *Paedocypris* spp. with SL = 8 mm. (**B**,**E**) Geographical maps with the distribution of both species and peatland forests highlighted in red. (**C**,**F**) Idiograms of both species’ chromosomal complements are dominated by large bi-armed chromosomes, revealing their probable fusion origin.

Despite their large geographical and phylogenetic distance, it is notable that both *N. anduzei* and *Paedocypris* spp. (Cypriniformes, Cyprinidae) share similar features (Figure 4). *Paedocypris* is considered to be the world’s smallest vertebrate, maturing at 8 mm SL [80] with a striking sexual dimorphism and inhabiting the highly acidic blackwaters of the peat swamp forests in Southeast Asia [81]. Peat swamp forests were also found in the Brazilian territory, especially in the middle and upper portions of Negro River [82], where *N. anduzei* is distributed. *Paedocypris* genus is a separate lineage within Cypriniformes (sometimes recognized as Paedocyprinidae) [83,84,85] that is represented by a group of species having reduced chromosome number when compared to other related species (2n = 50) [19,81,86,87]. It is considered that chromosome fusions of the Robertsonian type and genome size reduction were responsible for the diploid chromosome number reduction in the species [19,20]. Available studies indicate that several *HOX* genes have been lost in genomes of *Paedocypris* species, which may be responsible for their miniaturization process [20]. The same hypothesis could explain the reduced chromosomal number in pencil fishes, although research on *HOX* gene content and genome size should also be performed. Interestingly, although *Paedrocypris* spp. present a nuclear DNA reduced in size, mitochondrial DNA was not affected and has ~17 kb, which fits in the range known for a typical vertebrate genome of 15–20 kb [85].

Decreased diploid chromosome number (2n) has also been reported in other fish species that are not necessarily miniature ones. Some examples of low 2n and karyotypes dominated by bi-armed chromosomes include the Asian swamp eel *Monopterus albus*, which has two karyomorphs (2n = 18 and 2n = 24) that coexist in peat swamp forests in Thailand, in contrast to its congeneric and co-familiar species, which have karyotypes with 2n greater than 42 [86]. Therefore, miniaturization, in some cases, is more likely related to species’ population dynamics (particularly, small population sizes) because these species inhabit some marginal and/or extreme habitats [3,80,88,89]. In salmonid fishes, [89] hypothesized a link between reduced genetic diversity and the anadromous life history and/or intralacutrine speciation of some species, in which chromosome rearrangement fixation was favored due to a reduction in breeding population size. As a result, [90] hypothesis of selection for reduced recombination may have played a role in major genome reorganization, likely not exclusively in salmonid fishes. However, how could a specific type of environment have an impact on the miniaturization process? Peat swamp forests are already known as ecosystems that promote the establishment of small-sized species while inhibiting the presence of large-sized ones [80]. As a result, the species that reside there face significant selection pressure, resulting in the prevalence of tiny ones, including miniatures. Furthermore, because many individuals might be separated in small lakes or marginal habitats, all the aforementioned species form small populations with small population sizes that facilitate the fixation of chromosomal rearrangements [88]. For the *Paedocypris* spp., the convergent evolution of similar phenotypes to environmental selection was considered to be the main evolutionary process that generates that miniature species [84]. More research on the functional genetics, ecology, and evolution of miniature fishes is needed to better understand the mechanisms that originate and perpetuate such fish diversity.

## 5. Conclusions

Working with miniature fishes is obviously a tough task, but research involving such species is making substantial progress, particularly in chromosomal studies, once breakthroughs in cytogenetic processes overcome old technological constraints. Here, we analyzed the miniature species *N. anduzei,* and besides clarifying its main chromosomal features, we discovered another example of severe 2n reduction in fishes caused by numerous centric fusions, representing the second case in the genus of such extreme chromosomal reshuffling. Is it a coincidence that every miniature fish species examined so far showed such a large drop in 2n? The association between such 2n reduction and miniature body size among fishes is intriguing and deserves further investigation.

## Data Availability

The data presented in this study are available on request from the corresponding author.
